# Maximum entropy networks show that plant–arbuscular mycorrhizal fungi associations are anti‐nested and modular

**DOI:** 10.1111/nph.70694

**Published:** 2025-11-09

**Authors:** Sobia Ajaz, Nida Amin, Álvaro López‐García, Henry Birt, Mariona Pajares‐Murgó, Luisa Lanfranco, José L. Garrido, Julio M. Alcántara, Matthias C. Rillig, David Johnson, Tancredi Caruso

**Affiliations:** ^1^ School of Biology and Environmental Science University College Dublin Dublin D04 V1W8 Ireland; ^2^ Department of Soil and Plant Microbiology and Symbiotic Systems Estación Experimental del Zaidín, Spanish National Research Council (CSIC) Madrid 18008 Spain; ^3^ Department of Earth and Environmental Sciences University of Manchester Michael Smith Building Manchester M13 9PL UK; ^4^ Lancaster Environment Centre Lancaster University Bailrigg Lancaster LA1 4YQ UK; ^5^ Department of Animal Biology, Plant Biology and Ecology University of Jaén Jaén 23071 Spain; ^6^ Interuniversity Research Institute for the Earth System in Andalusia University of Jaen Jaén 23071 Spain; ^7^ Department of Life Sciences and Systems Biology University of Turin Viale P.A. Mattioli 25 Turin 10125 Italy; ^8^ Freie Universität Berlin Institute for Biology, Plant Ecology Altensteinstr. 6 Berlin 14195 Germany

**Keywords:** maximum entropy bipartite networks, modularity, nestedness, network structure, null models, plant–AMF association

## Abstract

- There is uncertainty in whether there is a common pattern of nestedness and modularity in plant–arbuscular mycorrhizal (AM) fungi associations, partly because of limitations arising from the use of null models that randomly rewire the observed connections to test for non‐random patterns in the network.
- Here, we overcome these limitations by generating null association matrices using maximum entropy network modelling, and specifically the bipartite binary configuration model (BiCM) with degree distributions as soft constraints. This was used to test the hypothesis that nestedness and modularity are prevalent in plant–AM fungi associations.
- In contrast to past findings, we found most plant–AM fungi associations were anti‐nested and modular. This pattern was almost universal, being consistent across habitat types, multiple spatial scales, and multiple levels of plant node aggregation, from communities and species to populations. Anti‐nestedness can easily emerge from modularity when network patterns are determined by the identity of the plant and AM fungal nodes.
- Our findings emphasize the need for experiments that test the factors that cause the observed network structure and how that structure determines the function and stability of plant–AM fungi association networks.

## Introduction

High‐throughput sequencing techniques have shown that arbuscular mycorrhizal (AM) fungi form intricate, multi‐species communities associated with plants (Öpik *et al*., 2009; Hart *et al*., 2015; Morgan & Egerton‐Warburton, 2017; Luo *et al*., 2020). These techniques have also demonstrated that AM fungi form dynamic and complex associations with plants at multiple scales and levels of biological aggregation (e.g. taxonomic, local vs metapopulations, individual plant species, fungal strains). The associations are influenced by environmental factors (Melo *et al*., 2019; Lu *et al*., 2022; Han *et al*., 2023), geographic location (Ujvári *et al*., 2021), and various biological factors such as the developmental stage of the host plant (Liu *et al*., 2023). There is, therefore, increasing interest in how AM fungi and plant communities come together to form ecological associations (Moora & Zobel, 1996; Horn *et al*., 2017; Wagg & McKenzie‐Gopsill, 2023; Ahammed & Hajiboland, 2024; Marrassini *et al*., 2024), and to understand the benefits that AM fungi confer on plants, including phosphorus (P) acquisition (Smith *et al*., 1997), resistance to diseases (Adeyemi *et al*., 2023; Kaur *et al*., 2023; Wahab *et al*., 2023), defence against herbivory (Babikova *et al*., 2013a,b, 2014), and the broader ecological services of soil carbon accumulation (Hawkins *et al*., 2023; Wu *et al*., 2024) and agricultural sustainability (Rillig *et al*., 2019). Besides benefits, plants and AM fungi also interact along a mutualism–parasitism continuum (Johnson *et al*., 1997), and along that continuum, individual AM fungal (AMF) species, populations, and strains can colonize numerous host plants (Sanders, 2002), which contributes to the complexity of these associations.

**Fig. 1:**
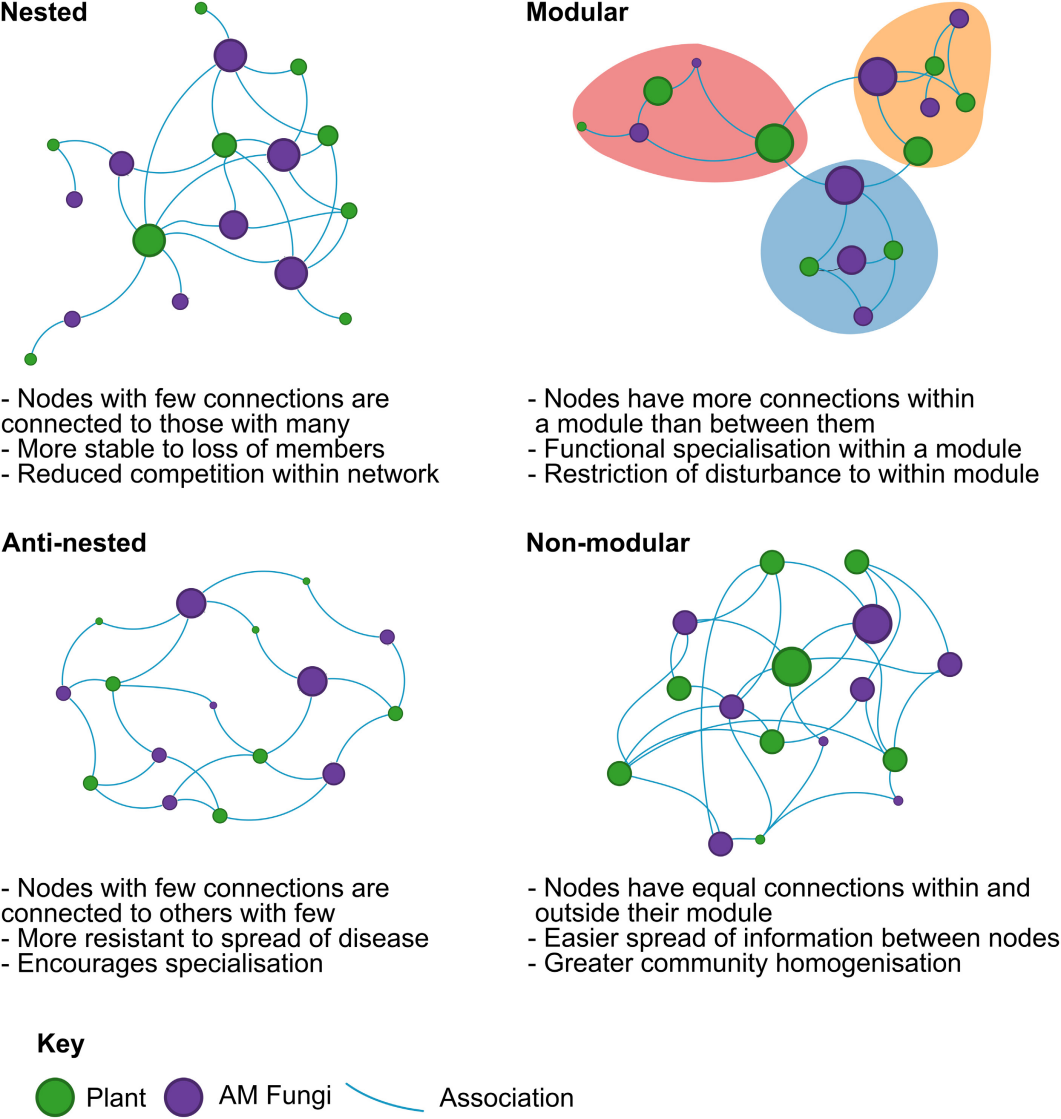
The two possible structures of plant–AM (arbuscular mycorrhizal) fungi association bipartite networks analysed in this work. There are two sets or layers of nodes (plant species/populations in green and AM fungal taxa in purple). The left panels illustrate nestedness and anti‐nestedness, the right panels illustrate modularity.

Such ecological complexity lends itself to the application of network analysis to describe and quantify patterns in the associations between plants and AM fungi at multiple levels of organization (population, species, community), which has led to several influential studies (Chagnon *et al*., 2012; Montesinos‐Navarro *et al*., 2012, 2019; Sepp *et al*., 2019; Garrido *et al*., 2023). These studies have demonstrated the potential of network approaches to reveal complex association structures and their ecological significance. Note that we prefer the more neutral word ‘association’ to ‘interaction’, given the complexity of inferring interactions from association matrices (Caruso *et al*., 2012), but also acknowledging that a number of those associations may also be interactions. Given the symbiotic nature (along the mutualism–parasitism continuum) of plant–AM fungi associations, a common way to model them is bipartite networks (Fig. 1), in which one layer represents AM fungi and the other represents plants, with each node typically, but not necessarily, representing a species (e.g. Bascompte *et al*., 2003; Bascompte & Jordano, 2007). In fact, the very first step to detect any pattern in a network is a decision on the level of taxonomic resolution at which the nodes should be defined, which is a well‐known point in the analysis of networks such as food webs (Martinez, 1993). However, this might be very difficult for AM fungi, given the complexity of their biology and taxonomy and in cases where AM fungi are identified using DNA sequencing. In the latter case, nodes may represent virtual taxonomic units (VTs), which are defined through molecular data and allow a finer resolution of fungal diversity (Davison *et al*., 2015). The plant layer, too, can be described across all levels of biological aggregation: The nodes may represent multiple populations of the same species (one node per population), with multiple species in the network. They can even represent an individual plant, with multiple individuals from multiple populations of multiple plant species, and with all of those levels of organization replicated across different conditions (e.g. control and treatments in manipulative experiments). In other words, there is no limit to the levels of biological aggregation at which the nodes can be defined, both for the plant and the AMF layer (Caruso *et al*., 2022), and network models can offer information at all of those levels (Neal *et al*., 2024).

Once bipartite network matrices describing plant–AM fungi associations are constructed at the desired level of biological aggregation, they can be analysed with tools from network science. The first goal of the analysis is the search for patterns in the network structure, for example, topological patterns linked to the degree distribution and mesoscale motifs, or also at the global scale or macroscale of highly aggregated properties such as connectance. Two other important goals are understanding the processes behind those structures and the functional implications of those structures (Newman, 2018). Here, we concentrate on the first goal and ask whether there are very common patterns in the structure of plant–AM fungi association networks or whether these patterns are context‐dependent and not generalisable. Some studies suggest that there are very common patterns (e.g. Chagnon *et al*., 2012; Montesinos‐Navarro *et al*., 2012). For example, nestedness has been proposed as a common feature of mutualistic networks (Bascompte *et al*., 2003; Bascompte & Jordano, 2007), including plant–AM fungi association networks (Chagnon *et al*., 2012; Montesinos‐Navarro *et al*., 2012). Nestedness describes subsets of associations in a network. For example, specialized AM fungi associate with a small number of plant species, and generalist AM fungi associate with a large number of plant species (Fig. 1). At the same time, the number of plant species associated with specialized AM fungi is a subset of the large number of plant species associated with generalist AM fungi. Clearly, real bipartite plant–AM fungi associations, as well as any bipartite ecological network, are never perfectly nested, and this generates the question of how to quantify nestedness and assess whether an association network can be defined as nested or not (Neal *et al*., 2024). Another feature that has been proposed as typical of plant–AM fungi associations is modularity (Montesinos‐Navarro *et al*., 2012; Garrido *et al*., 2023), in which species form groups, and the number of associations within a group is greater than that between groups (Newman, 2006, 2018). As for nestedness, the question arises of how modularity can be quantified to identify modules and assess whether a network is modular or not.

The quantification of network structures such as nestedness and modularity is the first step to move the field of research forward. Patterns in these network features can shed light on both the processes that generate the network and the dynamic or functional aspects of the network. For example, theory shows that certain network structures can enhance the stability of the populations represented by the network nodes, promoting resilience against environmental fluctuations and species loss (Pimm, 1984; Stouffer *et al*., 2005; Montoya *et al*., 2006; Allesina & Tang, 2012). Network approaches are thus a useful tool to investigate ecological and evolutionary dynamics of association patterns, including plant–fungal associations (Toju *et al*., 2014). However, the quantification of network structure is itself challenging. For example, intuitively, one might expect that the specialized associations implied by modularity could lead to anti‐nestedness rather than a set of specialist species nested in a set of generalist ones. Yet this is not necessarily the case (Valverde *et al*., 2020), and some studies show that correlations between nestedness and modularity can be mediated by other, basic, network features such as connectance (Fortuna *et al*., 2010); high connectance leads to modular but anti‐nested sets, and low connectance leads to the opposite. Similarly, the relationship between nestedness and more dynamic properties, such as stability, can also be complex, with studies suggesting either positive (Bascompte & Jordano, 2007) or negative (Staniczenko *et al*., 2013) effects. Other authors have also suggested that plant–AM fungi association networks can be anti‐nested and non‐modular (Encinas‐Viso *et al*., 2016), which was interpreted as a ‘lack of substantial structure’. That would imply that factors conferring stability to these associations should be researched by investigating other structural aspects of the association (Toju *et al*., 2015).

To address these uncertainties, we ask whether there is any very common pattern of nestedness and modularity in plant–AM fungi associations. We emphasise that the model chosen to describe and quantify plant–AM fungi association networks is essential. Current techniques rely on null models (Harvey *et al*., 1983; Gotelli & Ulrich, 2012), which are mostly derived from numerical algorithms that ‘randomly’ rewire the observed adjacency matrix that codes the information of who is associated with whom. The rewiring of the observed matrix is constrained to features such as the marginal sums (by rows, columns, or both) of the matrix so that the random matrices retain some key aspects of the observed data while randomising all other features related to the identity of the nodes (Bascompte *et al*., 2003; Blüthgen *et al*., 2007; Dormann *et al*., 2009). The choice of constraints in the null model rewiring algorithm, as well as in any null model technique, is of fundamental importance for detecting a structural pattern in the network because that choice represents the model assumptions, which affect the results (Neal *et al*., 2024). We argue that in the case of existing plant–AM fungi association datasets amenable to bipartite network analysis, the choice of the constraint and how they are modelled should reflect the fact that there is a degree of uncertainty in the measurements of plant–AM fungi associations, which propagates to the constraints used to create the null models. The uncertainty, typical of ecological datasets, is due to a combination of experimental errors such as those inherent in molecular approaches, but also natural fluctuations in populations and associations, both in space and time at multiple scales (Moora *et al*., 2011; Sánchez‐Castro *et al*., 2012; Davison *et al*., 2015, 2018; Polme *et al*., 2016; Zhao *et al*., 2021). Yet, the fact that there is uncertainty does not invalidate the dataset itself because, as a matter of fact, there are well‐documented associations and interactions between plants and AM fungi, and the existing datasets seek to approximate those associations and interactions in the form of a network matrix. The goal then becomes the estimation of structures in that matrix, taking into account unavoidable experimental errors and natural variability in the matrix entries.

Classic rewiring null models are very well benchmarked for certain types of ecological datasets, but are neither the only option nor the best option when there are natural fluctuations and experimental uncertainty in the data used to constrain the null models, which, in our opinion, is almost always the case for ecological networks (Caruso *et al*., 2022; Neal *et al*., 2024). In this work, we propose that the null model best suited to plant–AM fungi association datasets should account for fluctuations in the constraints. That can be done using maximum entropy network models with soft constraints (Squartini & Garlaschelli, 2017) rather than the fixed, so‐called ‘hard’ constraints of classic rewiring models, which assume no or negligible uncertainty in the constraints. In this work, we specifically apply the maximum entropy bipartite configuration model (BiCM) with soft constraints (Saracco *et al*., 2015). We concentrate on the binary version (presence–absence data) to work on the co‐occurrence matrix that associates plants and AM fungi, and the degree sequence constraint (i.e. the number of links to each node), node by node. In classic ecological null models with hard constraint (Neal *et al*., 2024), the rewiring of the observed matrix is most often constrained to the observed number of associations, node by node. That way, of all possible randomised matrices, only those that respect the degree sequence (or matrix marginals) exactly are accepted. That construction equates to a uniform probability distribution (Neal *et al*., 2024). A desired number of null random matrices (say 999) need to be generated with algorithms that simulate the sampling of the ensemble by reconstructing each random matrix. That is needed because it is impossible to actually generate the set of all possible matrices for moderately large and realistically heterogeneous datasets and then randomly sample a desired number of matrices from the full set of all possible ones. That fundamental limitation causes major statistical issues as it is hard or impossible to guarantee an unbiased sampling of the ensemble, as discussed thoroughly in the more technical literature (e.g. Squartini & Garlaschelli, 2017). In the case of BiCM, random matrices are not constructed by rewiring. Rather, given the degree sequence, we search for the probability distribution of *p*_*ij*_, which is the probability that node *i* in one layer (say the plant one) is connected to node *j* in the other layer (say the AMF one), for every possible pair *ij*. The model outcome is, therefore, a probability matrix. This probability matrix or distribution is found by searching for the one that maximises the entropy of the network, subject to two constraints, as we explained in more detail in the method section. As described in the methods, only the average degree of each node across the random matrices will be equal to the observed degree. That is why the constraint is defined as ‘soft’, compared to the ‘hard’ constraint of rewiring models. The various realisations of the ensemble of random matrices of the BiCM represent fluctuations around the observed configuration (Caruso *et al*., 2022). The statistical virtues of this modelling approach and its advantages over the classic ones have been demonstrated elsewhere (Squartini & Garlaschelli, 2017; Parisi *et al*., 2020). Here, we stress that there are no right or wrong models; we only have assumptions about models that fit different situations. We propose here that the best benchmarking null model to assess whether plant–AM fungi associations are nested and modular is the BiCM in reason of the soft constraints, the statistical robustness (unbiased estimates and maximised randomness), and the computational efficiency (a Python package as well as a Python code based on that package already exist to fit this model, but here we also offer a new R code to fit the model, also including some useful data wrangling functions that facilitate the analyses).

We analysed 36 publicly available plant–AM fungi association datasets derived from different ecosystems and at varying levels of biological organization (individual, population, and species) using the maximum entropy BiCM. We quantified nestedness and modularity of observed and null model ensemble matrices to test if nestedness and modularity in plant–AM fungi association networks across multiple scales and levels of biological organisation are common and widespread. We then interpreted the implications of results in terms of community assembly and dynamics (e.g. stability and resilience) to suggest how the field of research can move forward in the light of emerging methods of network analysis and the results they are offering.

## Materials and Methods

### Datasets

The datasets used in the current study were obtained from open access peer‐reviewed publications searched in Web of Science, Scopus, and Google Scholar as per the documentation in the Prisma Flow Diagram (Supporting Information Fig. S1). We also explored all the studies available in the MaarjAM database (https://maarjam.ut.ee) (Öpik *et al*., 2010). In summary, for our searches in Web of Science and Google Scholar, we used the keywords ‘plant‐AMF association’, ‘AMF abundance’, and ‘AMF sequencing in plants’. We retrieved the results and removed too small datasets defined as AMF nodes < 5 and at least one plant species (datasets with only one plant were considered if we had other factors in the experiment that could be used as plant nodes, like treatments/genotypes/time points, etc.). The 36 different studies were finalised based on the availability of data of AMF species in plant roots or plant root‐associated rhizosphere soil. AMF Virtual Taxon (VTs)/Operational Taxonomic Unit (OTUs) were treated as nodes unless species‐level identification was given by the authors. (The VT/OTUs were linked to AMF classification through MaarjAM in the datasets where VT/OTU information about fungi was not available in the respective paper.) Some of the datasets were subsets for AM fungi when other groups of fungi were involved in the study, or where AM fungi were studied in both plant and soil samples. In terms of selection criteria to finalise the choice of the datasets (see Prisma Flow Diagram in the Fig. S1), the key principle was that we looked for studies in which the AMF strain, population, or more generally taxon datum (most commonly OTU or VT) was associated with a plant‐level datum, either individual, population, or species (for plants, species was the most common case by far). We thus relied on the authors' definition of the association between the AM fungus and the plants, and we used the word ‘association’ to signify that the definition is broad, covering association at a range of scales, from the broadscale of meta‐networks to the very local associations between individual plants and the AM fungi found in their roots or rhizosphere soil (as described later in more detail). Also, given this approach, we did not undertake bioinformatic analysis of the original sequences of the AMF datasets, but instead relied on the original associations. We therefore considered studies providing association matrices rather than rebuilding new association matrices. This is important because we removed one potential layer of confounding factors (e.g. different bioinformatic approaches and definition of AMF taxa) in the comparison of our results with the results previously published. For the abundance data (e.g. number of reads per OTU) were converted to presence–absence data, and for the same reasons explained above (consistency with the published association matrices), we did not apply any threshold criterion in terms of the level of abundance required to assign a link between an AMF node and a plant node. In other words, as long as the original, published matrix defined an AMF node as associated with a plant node (i.e. a nonzero entry), we accepted that published datum as a valid link.

### Range of conditions encompassed by the datasets

The datasets chosen possessed a range of AMF VTs (8–277) and plant species (1–245) (Table 1). In the dataset with one plant species, as well as in others, the plant layer consisted of multiple individuals or populations of the same plant species, in which case the plant layer nodes would represent individuals or populations, depending on the study. This approach enabled us to explore how AMF associations might be structured within populations and individuals of the same plant species as well. The sequencing methods used in the chosen studies also varied. There were 20 studies based on cloning and sequencing, ranging from 2009 to 2018. The other 16 studies were based on Illumina MiSeq/HiSeq/Novaseq, rDNA sequencing, and 454 sequencing platforms. The datasets comprised plants from diverse vegetation categories and habitats, including grasslands, cropland, forests, and deserts. Various studies considered here were conducted at multiple spatial scales and in some cases with multiple scales within the same study. To explore the role of scale we thus considered three levels: global to very broadscale lists of species that may be aggregating plants from different biogeographic regions; more local datasets including the same species but with populations or individual plants under different environmental conditions (e.g. experimental treatments); and very local datasets representing physical biological communities as they may be operating on a small scale. For example, a single plant species study was of this kind, in which case network analysis can reveal the degree of specialized association in AM fungi across the plant species populations. More generally, the case of very local datasets representing physical biological communities can be considered a realization of associations that are known to be possible given the meta‐network of associations in the relevant regional pool of species (Rollin *et al*., 2024). At the same time, the complementary view is also possible, that is, the meta‐network can be constructed from the collection of local networks, which is what is typically done in the inference of metacommunity dynamics and metapopulation dynamics (Hanski, 1999; Leibold *et al*., 2004). More operationally here, to further address in detail the potential effect of data aggregation (scale‐wise and biologically in terms of multiple populations of the same plant species), we focused on a specific dataset (Garrido *et al*., 2023, available at Garrido Sánchez *et al*., 2024) to validate observed patterns at multiple levels of data aggregation within a single study. This dataset was collected in two different Mediterranean mountain systems in southern Spain, separated by *c*. 100 km. In each system, the plant–AM fungi assemblies were characterized by repeatedly sampling plant roots of several species in different locations of the same ecosystem (between 6 and 24 samples per plant species and mountain system; see Garrido *et al*., 2023 for details). Therefore, to further test the impact of data aggregation on the detection of structural patterns and interpretation of those patterns, we considered the following levels of aggregation: plant species meta‐network (all locations lumped together), plant species but with the two main mountain systems separately, and population of the same plant species across multiple locations.

**Table 1:** Plant–arbuscular mycorrhizal fungal (AMF) association metadata of the datasets considered in this work.

Study	No. of plants	No. of AMF species/OUT/VT	Sequencing methodology	Plots/replicates/sampling units (*n*)
Galván *et al*. (2009)	1	14	rDNA sequencing	6
Öpik *et al*. (2009)	10	48	454 sequencing	10
Tchabi *et al*. (2009)	1	49	Cloning and sequencing	12
Wilde *et al*. (2009)	3	24	rDNA sequencing	7
Alguacil *et al*. (2011a)	6	12	Cloning and sequencing	12
Alguacil *et al*. (2011b)	4	8	Cloning and sequencing	4
Baar *et al*. (2011)	5	23	Cloning and RFLP	6
Davison *et al*. (2011)	11	40	Cloning and sequencing	11
Isobe *et al*. (2011)	1	24	Cloning and sequencing	10
Liu *et al*. (2011)	2	16	Cloning and RFLP	10
Moora *et al*. (2011)	1	71	454 sequencing	14
Sasvári *et al*. (2011)	1	33	Cloning and sequencing	6
Wang *et al*. (2011)	3	23	Cloning and sequencing	9
Alguacil *et al*. (2012)	3	20	Cloning and sequencing	3
Sánchez‐Castro *et al*. (2012)	5	37	Cloning and sequencing	5
Merckx *et al*. (2012)	33	56	Cloning and sequencing	33
Guo & Gong (2014)	17	22	Cloning and RFLP	17
Li *et al*. (2014)	2	29	Cloning and RFLP	16
Davison *et al*. (2015)	245	247	454 sequencing	247
Dieng *et al*. (2015)	3	13	Cloning and sequencing	17
Grilli *et al*. (2015)	1	59	454 sequencing	3
Varela‐Cervero *et al*. (2015)	5	81	Cloning and sequencing	5
Moora *et al*. (2016)	1	47	454 sequencing	5
Polme *et al*. (2016)	5	38	Cloning and sequencing	5
Campos *et al*. (2018)	3	92	Illumina MiSeq	30
Davison *et al*. (2018)	218	277	454 sequencing	277
Muneer *et al*. (2019)	3	52	Cloning and sequencing	3
Higo *et al*. (2020)	1	148	Illumina MiSeq	6
Lara‐Pérez *et al*. (2020)	1	21	Illumina MiSeq	7
Deepika & Kothamasi (2021)	12	18	Cloning and sequencing	35
Feng *et al*. (2021)	2	24	Cloning and sequencing	2
Zhao *et al*. (2021)	10	17	Illumina HiSeq	67
Fernández *et al*. (2022)	2	16	Illumina MiSeq	96
Garrido *et al*. (2023)	18	87	Illumina MiSeq	63
Djotan *et al*. (2023)	2	321	Illumina MiSeq	2
Wang *et al*. (2024)	12	207	Illumina NovaSeq	59

### Network modelling – metrics

Bipartite networks with two sets of nodes – plant and AM fungi – were used to describe plant–AM fungi associations. The abundance (based on what was originally reported in the primary studies) of each AM fungus was converted to a binary matrix (0 or 1 for absence or presence, respectively). The Bipartite R package (Dormann *et al*., 2009) was used to compute the metrics of nestedness and modularity using the network‐level and computeModules functions. In these functions, nestedness was measured with the nested overlap and decreasing fill (NODF) as per Almeida‐Neto *et al*. (2008). Modularity was measured using the classic approach originally proposed by Newman (2006) and applied to the bipartite case using Beckett's algorithm, which is available as one of the modes of the computeModules function in the R Bipartite package (Beckett, 2016). The approach uses a configuration model to maximize the modularity metric Q. The maximum value of the metric is achieved for the partition of the graph into groups of nodes (the modules) that maximize the number of connections within each module relative to the configuration model.

### Network modelling – the maximum entropy BiCM as null model

We used the maximum entropy bipartite binary configuration model (BiCM) as a null model to assess the level of nestedness and modularity in the AM fungi–plant association networks. The model revolves around two key ideas. First, we search for *p*_*ij*_, which is the probability of connection between a node in the fungal layer (say *i*) and one in the plant layer, say *j*. Second, the set of probabilities for every possible pair *ij* forms a probability distribution, and the one to use is the one that maximises the entropy metrics (Squartini & Garlaschelli, 2017) subject to the null model constraints. The constraints are aspects of the observed data that need to be preserved to formulate a meaningful null model. The constraint, in our case, is the degree sequence, which is the number of observed links to each node, node by node (in other words, the row and column marginals). Different from classic rewiring algorithms, the constraint is not to be met exactly by the random matrices of the null models. Indeed, it should not, because there is uncertainty or fluctuations in the degree sequence itself. The constraints should therefore be met just on average by the collection of random matrices in the null model ensemble. For the general case, the key formulas are:

(Eqn 1) $$S\left(P\right)\equiv -\sum_{E}P\left(E\right)\log_{e}P\left(E\right)$$

(Eqn 2) $$\left\langle C\right\rangle =\sum_{E}C\left(E\right)P\left(E\right)=C^{*}$$

In which Eqn 1 is the entropy of the network matrix **E**, and C* in Eqn 2 is the constraint as observed in the matrix (in our case, the degree sequence, a vector). In our approach, we search for the expression of the probability *P*(**E**) that maximises the entropy metric *S*. As shown in Squartini & Garlaschelli (2017) and references therein, the specific solution for the general, binary configuration model in which the network **E** is represented by the adjacency matrix **A** (with elements *a*_*ij*_, which code for the network links with 0 or 1) is

(Eqn 3) $$P\left(A|\theta \right)=\prod_{i}\prod_{j<i}{\;p}_{\mathrm{ij}}^{a_{\mathrm{ij}}}{\left(1-p_{\mathrm{ij}}\right)}^{1-a_{\mathrm{ij}}}$$

In Eqn 3, it can be shown that the probability $p_{\mathrm{ij}}=\frac{x_{i}x_{j}}{1+x_{i}x_{j}}$. This is the correct form of the connection probability between nodes *i* and *j*, with $x_{i}\equiv e^{-\theta_{i}}$ being a conveniently transformed Lagrange multiplier (the θ), which comes from the procedure of entropy maximisation. The method of Lagrange multipliers is indeed used to find the probability *P*(**E**) that maximises the entropy *S*(*P*), which is a classic statistical mechanics recipe. The knowledge of the functional form of $P\left(A|\theta \right)$ allows the calculation of the expected degree $\left\langle k_{i}\right\rangle$ as a function of the transformed Lagrange multipliers. This means that the probability can now be estimated if we equate the expected degree $\left\langle k_{i}\right\rangle$ to the empirical degree $k_{i}\left(O\right)$, where **O** is the observed matrix. This equality is the constraint enforced as an average. Specifically:

(Eqn 4) $$\left\langle k_{i}\right\rangle =\sum_{i\neq j}\frac{x_{i}x_{j}}{1+x_{i}x_{j}}=k_{i}\left(O\right)\;\forall i.$$

The values of $x_{i}$ solving the above equations also coincides with the values that maximise the log‐likelihood $L\left(\theta \right)$. Therefore, we can estimate the expected *p*_*ij*_ from the degree sequence by combining maximum entropy and maximum likelihood. That ensures an unbiased estimate, which is a major statistical virtue of the model. The extension of this model to the bipartite case is straightforward, as it simply involves a reparameterisation that considers the two sets of nodes in the two layers (e.g. plants and fungi) of the bipartite graph (Saracco *et al*., 2015), with links possible only between the two layers, and never within layers. The probability of connection between node *i* in the plant layer *x* and node *j* in the fungal layer *y* now becomes

(Eqn 5) $$p_{\mathrm{ij}}=\frac{x_{i}y_{j}}{1+x_{i}y_{j}}$$

with *x*_*i*_ and *y*_*j*_ as the new parameters to be estimated for every combination node *i* and node *j* between layers *x* and *y* (say plants and AM fungi in our case).

The estimation of all the *p*_*ij*_ returns a matrix of the probability of connection. This is effectively a probability distribution that can be sampled to draw a particular, realised matrix. The null model ensemble consists of any desired number of these matrices, say 999 or 9999. This model can be fitted using either the original Python code (see Caruso *et al*., 2022) or with the R code (Notes S1 that we provide in the supporting information together with an exemplar dataset file Dataset S1(bi_species).csv, which is a subset derived from the dataset in Campos *et al*., 2018, and on which the code can be run directly to generate: the probability matrix with the *p*_*ij*_ and an ensemble of 999 random matrices. These matrices will look similar to the observed one in terms of degree sequence, but the position of the links would be fully randomised, subject to the constraint that nodes that were observed to be highly connected will, on average, be highly connected, and vice versa. Specifically, the average of all the random realisations is such that the average degree sequences will exactly equal the observed degree sequences, as per Eqn 4. This can be verified numerically to check that the model fit was successful. The ensemble of the null model matrices represents fluctuations around the average matrix, which is assumed to be equal to the observed matrix, but only for the degree sequence, or any other properties that depend on that. Otherwise, the matrices are completely random and, indeed, maximally random according to the maximum entropy principle (Squartini & Garlaschelli, 2017).

Therefore, any deviation of the observed matrix from the distribution of the null model matrices implies that aspects of species identity not captured by the constraint (the degree sequence in the BiCM) significantly shape network structure. Specifically, the ecological interpretation of the BiCM is that it fully randomises species identity, or more generally node identity, while preserving the number of associations for each species or node. That model is, therefore, a full alternative to classic ecological null models that use the degree sequence as a constraint but randomise the observed links through a hard constraint and rewiring algorithms to generate a probability null distribution (Neal *et al*., 2024).

To compare the observed levels of nestedness and modularity to the null model and make a statement on whether plant–AM fungi associations are nested, modular, or both, we computed nestedness and modularity for each observed matrix. We then fitted the null model to every dataset and calculated nestedness and modularity for the resulting null model matrices (999). Finally, the *Z*‐score of each of the two metrics was computed as (NODF_observed_ − NODF_null model_)/SDNODF_null model_ for nestedness and (Q_observed_ − Q_null model_)/SDQ_null model_ for the modularity metrics. In both cases, 999 sampled random matrices were used to compute the NODF_null model_ and Q_null model_, which, respectively, were the average nestedness and modularity of the null model ensemble. We computed the corresponding SD of those averages (i.e. SDNODF_null model_ and SDQ_null model_).

The *Z*‐score measures the deviation of the observed metric from the null model distribution of the metric. For example, if the *Z*‐score of nestedness is negative, the network is anti‐nested relative to the average of the null model, while if positive, it would be nested. In the case of modularity, a negative *Z*‐score would result in a non‐modular network, while a positive one would result in a modular network. In other words, our descriptive definition of a nested/anti‐nested and modular/non‐modular network is relative to the baseline of the null distribution. The classic expectation of plant–AM fungi associations is that they form nested and modular association networks, and we thus tested the collection of datasets for this expectation. For simplicity, we describe a network as nested/anti‐nested and modular/non‐modular on the basis of the sign of the *Z*‐score. But to answer our main question in terms of a formal test, about whether an AM fungi–plant association network is nested and modular, we computed the null model *P*‐value for both metrics (Gotelli & Ulrich, 2012; Caruso *et al*., 2022) under the null hypothesis that the observed deviation, quantified by the *Z*‐score, is a random fluctuation from the null distribution. The concept of the null model *P*‐value is the classic one: A structural pattern can be considered statistically robust if the observed deviation from the average of the null model has a small probability of occurrence (in our case, we chose the widely used *P*‐value of 0.05).

Finally, to visualize network structures, we utilized the Gephi software (v 0.10.1). Network topology was arranged using the ForceAtlas2 layout algorithm. Within the resulting graph, nodes were scaled according to their degree (number of connections). Following export from Gephi, specific modules of interest were manually highlighted using polygon overlays created in Inkscape (v.1.3).

## Results

### Nestedness

NODF ranged from 6.33 to 69.19 (Table S1), where the minimum value was found for Merckx *et al*. (2012) and the maximum value was in Öpik *et al*. (2009). The values of NODF_observed_ were lower than the mean and median of NODF_null model_ in all except four datasets (Alguacil *et al*., 2012; Grilli *et al*., 2015; Higo *et al*., 2020; Djotan *et al*., 2023), where NODF_observed_ was higher. Standard deviation and variances are summarized in Table S1. The *Z*‐score ranged from 3.27 (nested) to −7.09 (anti‐nested), illustrated in Fig. 1. A total of 28 out of 36 datasets deviated from the null model (*P*‐value < 0.05), with 26 datasets anti‐nested and two (Grilli *et al*., 2015; Higo *et al*., 2020) nested (see Table S1 for a full summary of these results).

### Modularity

**Fig. 2:**
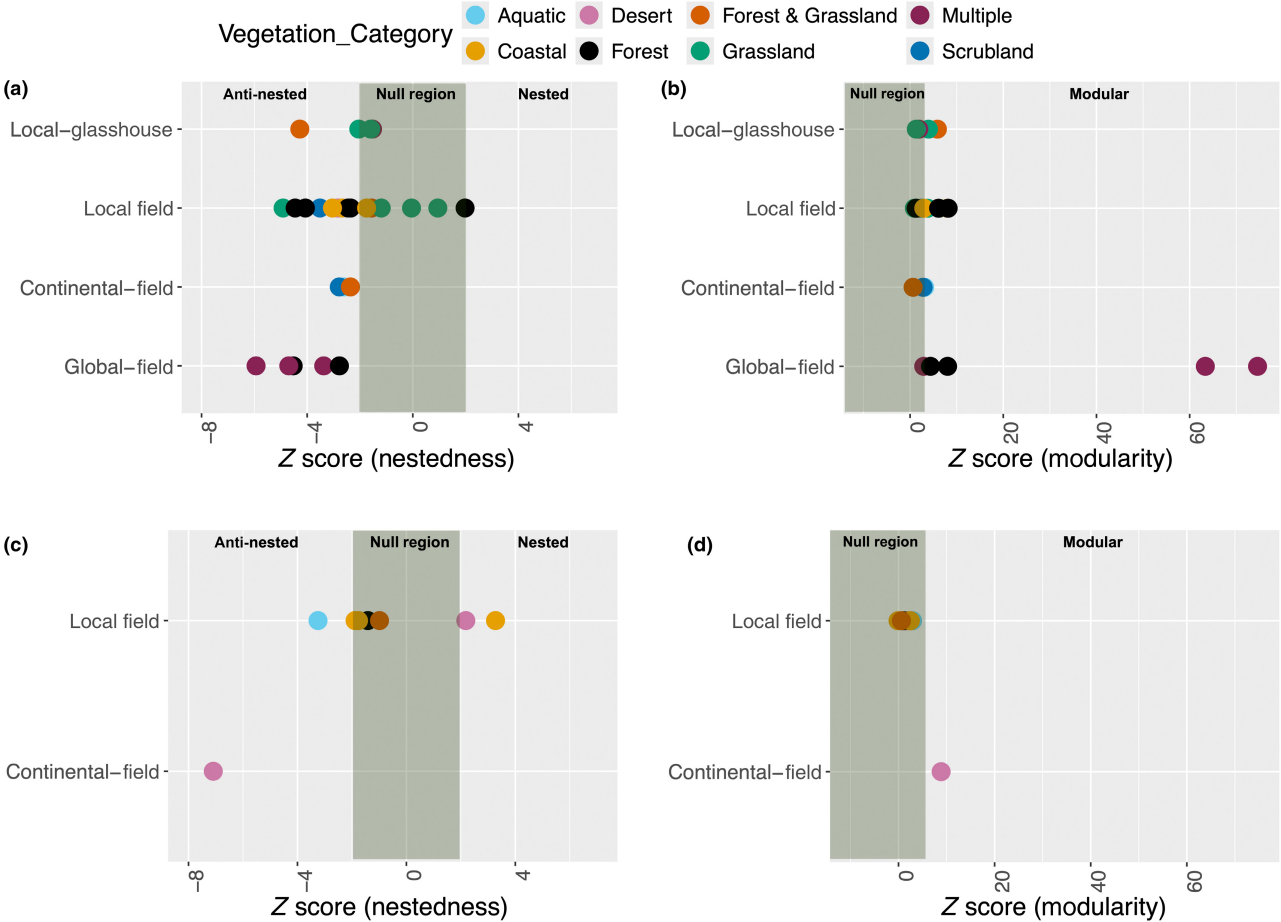
Nestedness (a, c) and modularity (b, d) at different scales of study (rows) and across different ecosystems (colours) to profile arbuscular mycorrhizal fungal communities in plants. (a) *Z*‐score nestedness is calculated based on N_observed_, average N_null model_, and SD of N_null model_ and (b) *Z*‐score modularity is calculated from M_observed_, average M_null model_, and standard Deviation of M_null model_. The green area represents a *Z*‐score of 0 ± 2 SE, which, under the assumption of a normal distribution of the metric under investigation, would represent *c*. 95% of the null distribution: In other words, an observed *Z*‐score falling within the green area is within the 95% confidence interval of the null model expectation for that metric. However, we used this interval in this figure only for visualisation purposes. For statistical tests on each dataset, we used the actual null model *P*‐values from the sampled distribution of each dataset‐specific model fit, without assuming a normal distribution of the metrics. For nestedness (a, c), a positive *Z*‐score to the right of the green area, that is > 2, implies a nested network (and so < −2 means anti‐nestedness, as observed for most datasets). For modularity (b, d), a positive *Z*‐score > 2 means a modular network, which is what was observed for all networks. The figure thus overall illustrates that almost all datasets were anti‐nested and all of them were modular. See Table S1 for the *P*‐values used in our analysis, and *Z*‐score value distribution.

Modularity ranged from 0.06 to 0.72 (Table S1), with the minimum value in maize (Higo *et al*., 2020) and the maximum value in wild herbs (Merckx *et al*., 2012). Out of the 36 datasets, 24 deviated from the null models (*P*‐value < 0.05), all showing larger modularity (Fig. 2). Depending on the datasets, the magnitude of deviation from the null model varied considerably. For example, the two largest datasets (Davison *et al*., 2015, 2018), encompassing a broad range of ecosystems and biogeographical regions, were highly modular with a *Z*‐score above 63 (*P*‐value < 0.001).

### Patterns of nestedness and modularity across vegetation categories

Four datasets with plants distributed across multiple vegetation categories were all anti‐nested (the largest *Z*‐score was −1.53, all *P*‐values ≪ 0.05) and modular (the smallest *Z*‐score was 1.88; all *P*‐values < 0.05). The same results were found in the two aquatic plant datasets from lakes (Baar *et al*., 2011; Moora *et al*., 2016), being highly anti‐nested (the largest *Z*‐score was −2.61, *P*‐value < 0.01) and modular (the smallest *Z*‐score was 2.92, *P*‐value < 0.001). The three datasets from coastal areas (Wilde *et al*., 2009; Guo & Gong, 2014; Deepika & Kothamasi, 2021) were anti‐nested and modular as well (smallest *Z*‐score NODF was −3.04; *P*‐value < 0.01 and largest *Z*‐score modularity = 8.05; *P*‐value < 0.01). The dataset from the desert (Wang *et al*., 2011) and the two from Mediterranean scrublands (Varela‐Cervero *et al*., 2015; Polme *et al*., 2016) were also anti‐nested and modular (*Z*‐score NODF < −2.78; and *Z*‐score modularity > 2.53; *P*‐value < 0.01; see Table S1 for a full summary of these results).

Nine datasets were from forests, and the nestedness *Z*‐score values for these ranged from 7.09 to 2.18, where seven datasets (Öpik *et al*., 2009; Moora *et al*., 2011; Merckx *et al*., 2012; Li *et al*., 2014; Zhao *et al*., 2021; Garrido *et al*., 2023; Wang *et al*., 2024) were significantly anti‐nested (*P*‐value < 0.01). The modularity *Z*‐score in these datasets ranged from 1.07 to 8.88, where six datasets (Moora *et al*., 2011; Merckx *et al*., 2012; Grilli *et al*., 2015; Zhao *et al*., 2021; Garrido *et al*., 2023; Wang *et al*., 2024) were significantly modular (*P*‐value < 0.04).

The cropland vegetation category consisted of four datasets (Galván *et al*., 2009; Isobe *et al*., 2011; Sasvári *et al*., 2011) that were anti‐nested (the largest *Z*‐score NODF was −1.76, *P*‐value < 0.05) and modular (the smallest *Z*‐score was 2.19, *P*‐value < 0.02). Only one dataset (Higo *et al*., 2020) was nested (*Z*‐score = 3.27, *P*‐value = 1), while its modularity did not depart from random expectations.

Plants from grassland were from seven different datasets, two of them (Campos *et al*., 2018; Muneer *et al*., 2019) anti‐nested (the smallest *Z*‐score was −4.91; *P*‐value < 0.001) and modular (the largest *Z*‐score was 3.95; *P*‐value < 0.001). The three datasets from both forest and grassland varied in their anti‐nested pattern and modularity scores: one (Fernández *et al*., 2022) was anti‐nested (*Z*‐score = −2.36; *P*‐value < 0.01) and modular (*Z*‐score = 6.00; *P*‐value < 0.01), while one dataset (Davison *et al*., 2011) was anti‐nested (*Z*‐score = −2.36; *P*‐value < 0.01), and the other (Liu *et al*., 2011) was modular (*Z*‐score = 2.40; *P*‐value < 0.01).

### Patterns of nestedness and modularity at different levels of aggregation of the same dataset

**Fig. 3:**
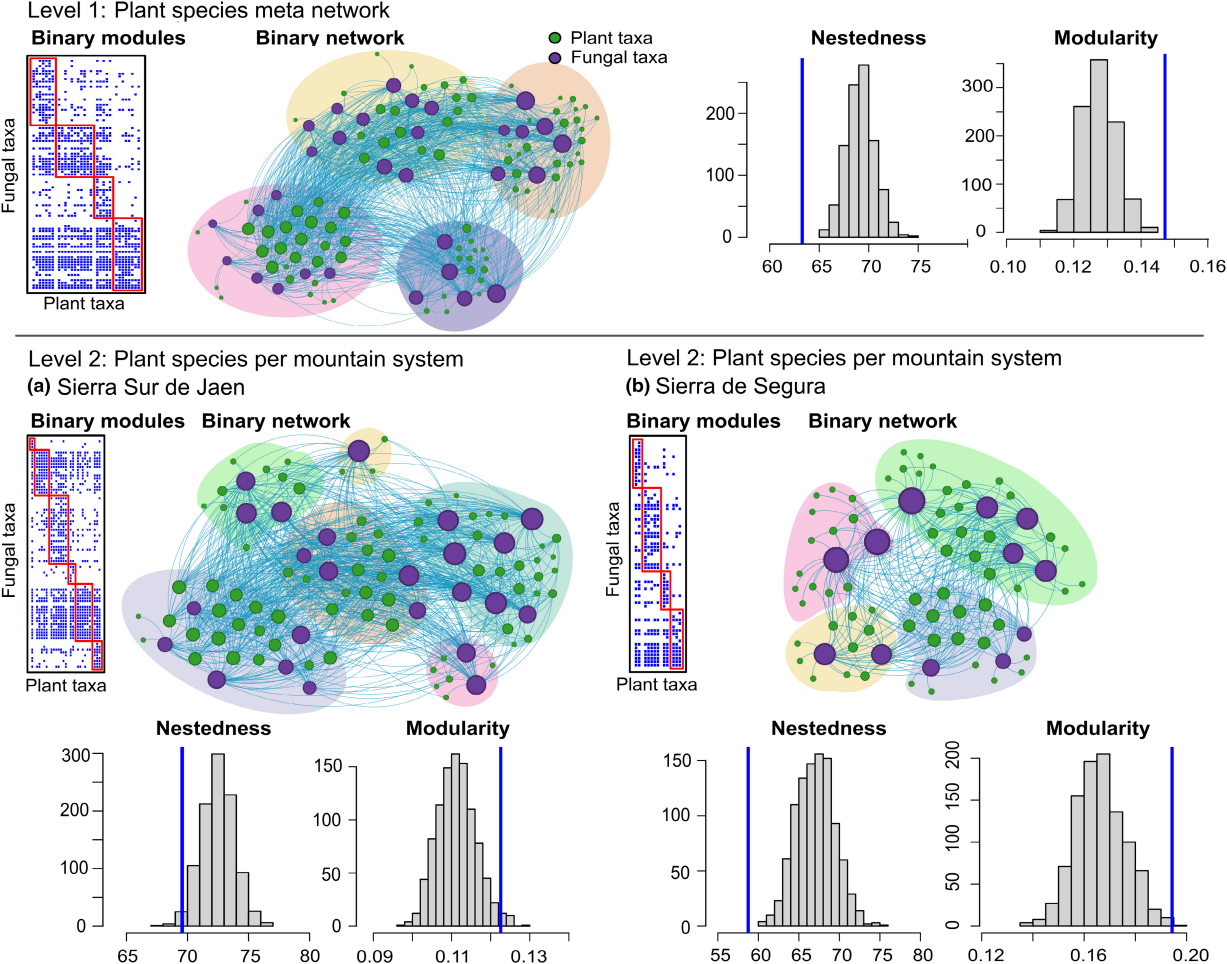
The aggregation of Garrido *et al*. (2023) datasets at three scales. The first level (upper panel, level 1) represents a meta‐network, where plant nodes represent species, regardless of location or habitat differences. The second level (lower panel, level 2) represents plant communities, with plant nodes representing species co‐occurring in each mountain system, with (a) Sierra Sur de Jaen and (b) Sierra de Segura. See Fig. 4 for a further level of aggregation. The figure shows both the matrix and graphic representation of the networks. The matrix is organized by the detected modules (red rectangles), with the blue dots indicating an association between a plant and an arbuscular mycorrhizal fungus. In the network graph, the modules are also highlighted by the coloured clouds surrounding the groups of nodes identified in the analysis as modules. The histograms report the results of the null model, with the histogram bars representing the frequency (*y*‐axis) of the network metrics (either nestedness or modularity; *x*‐axis) in the null model, and the vertical blue line representing the observed value of the same metric (on the *x*‐axis).

**Fig. 4:**
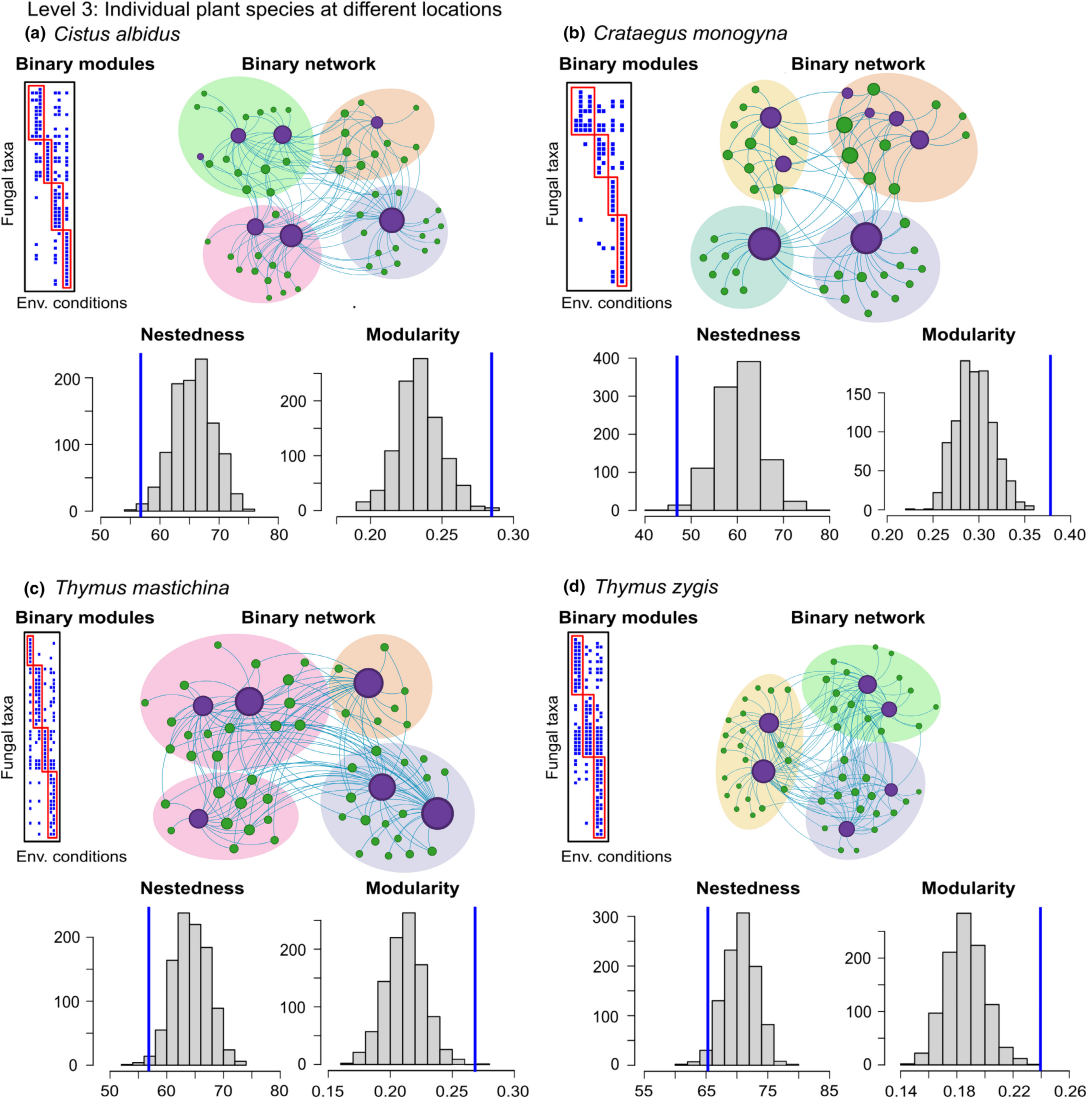
Third level of aggregation of Garrido *et al*. (2023) datasets (see Fig. 3 for the other two levels). Data were aggregated at the plant species level. (a) *Cistus albidus*, (b) *Crataegus monogyna*, (c) *Thymus mastichina*, and (d) *Thymus zygis*, with the plant node layer representing local populations of the species. The figure shows both the matrix (nestedness and modularity) and graphics (binary modules and binary network structure) of the network.

We initially pooled the whole Garrido *et al*. (2023) dataset by plant species, regardless of location or habitat differences, effectively constructing a meta‐network of all potential associations a plant species might have (Figs 3, 4). The aggregated dataset was found to be anti‐nested (*Z*‐score NODF = −4.10, *P*‐value < 0.05) and modular (*Z*‐score = 3.61, *P*‐value < 0.05). The second level of aggregation aimed at identifying local biological communities of plants and AM fungi effectively associated with specific geographic locations. This was achieved by building one adjacency matrix per mountain system (i.e. Sierra Sur de Jaen and Sierra de Segura). In both mountain systems, plant–AM fungi association networks were anti‐nested (*Z*‐score < −2.18, *P*‐value < 0.01) and modular (*Z*‐score > 2.42, *P*‐value < 0.01; Fig. 3). The third level of aggregation accounted for habitat/dispersal differentiation and local communities associated with different populations of the same plant species, and for that, we generated adjacency matrices for four individual plant species (*Thymus zygis*, *Thymus mastichina*, *Cistus albidus*, and *Crataegus monogyna*), where each population was treated as a different associating node (Fig. 4). All four networks were anti‐nested (the largest *Z*‐score was −2.03, *P*‐value < 0.02) and modular (the smallest *Z*‐score was 2.41, *P*‐value < 0).

## Discussion

We tested whether there was any common pattern in the level of nestedness and modularity of plant–AM fungi associations across multiple spatial scales and levels of biological aggregation of the plant nodes. Our results show consistent evidence of a common set of structural features, namely anti‐nestedness and modularity, found at all the spatial scales and habitat types, and also at multiple levels of biological aggregation. This result is to be explained taking into consideration that plants and fungi interact along a complex mutualism–parasitism continuum (Johnson *et al*., 1997) so that along that continuum, individual AMF species, populations, and strains can associate with numerous host plants (Sanders, 2002), and similarly, plants may host particular AMF species, populations, and strains (Kiers & Heijden, 2006). In fact, the degree of association between plant populations/species/communities and AM fungi can vary greatly in space and time at various scales (e.g. Vályi *et al*., 2016), and some evidence suggests that stochastic processes may also be a major driver of plant–AM fungi community assembly (Dumbrell *et al*., 2010; Lekberg *et al*., 2012) and may also shape interactions (Cirtwill *et al*., 2019; Parisy *et al*., 2024; Toju *et al*., 2024). That means that the number of species per sampled location or of AM fungi associated with a plant species or, vice versa, of plant species associated with an AM fungus will vary greatly in space but also over time for the same sampling point. The network null model should reflect that variation in the topological constraints, namely the degree sequence in our case. There are also unavoidable experimental errors such as those inherent to molecular methods and field sampling, which further complicate the quantification of network patterns. In light of this complexity, we propose that our network modelling strategy could detect consistent patterns by embracing the fluctuations we must expect in plant–AM fungi associations. Specifically, the fluctuations need to be modelled at the level of the null model constraints, node by node (Caruso *et al*., 2022), which in our case was the classic degree sequence.

Do the observed patterns align with or contradict the results obtained in previous works? First, and in line with the existing literature, we observed a high and commonplace level of modularity, which validates the notion that selectivity in plant–AM fungi interactions might result in specific community structures with sets of plant species or populations sharing relatively unique sets of AMF taxa (Vandenkoornhuyse *et al*., 2002, 2003; Mony *et al*., 2021). We also observed, however, that plant–AM fungi association networks are mostly anti‐nested rather than nested, which is contrary to some past observations for mutualistic networks but consistent with others (Toju *et al*., 2014, 2015; Encinas‐Viso *et al*., 2016). In general, nestedness has been proposed as a common feature of mutualistic networks (Bascompte *et al*., 2003; Bascompte & Jordano, 2007), including plant–AM fungi association networks (Chagnon *et al*., 2012; Montesinos‐Navarro *et al*., 2012). However, it has also been reported that plant–AM fungi association networks can be anti‐nested and indeed non‐modular (Encinas‐Viso *et al*., 2016), opening the possibility that a high level of nestedness might not be a feature of AMF associations. Indeed, while we did find some nested networks, we found that anti‐nestedness is much more common and coupled with a modular structure in the majority of the datasets we analysed. This finding supports the notion that networks with a strong community structure (e.g. highly modular) could be either nested or modular but usually not both, especially at a certain level of connectance (Fortuna *et al*., 2010). Almeida‐Neto *et al*. (2008) showed that different ecological processes can generate network structures that are less nested (i.e. anti‐nested) than expected from pure chance. Thus, anti‐nestedness should not be interpreted simply as the opposite of nestedness. Moreover, Almeida‐Neto *et al*. (2008) showed that common association structures (e.g. checkerboard) can show lower nestedness than expected by chance. Interestingly, various structures show a high degree of modularity: For example, a bipartite matrix with a checkerboard pattern can be rearranged into a two‐module structure. By definition, compartmented structures have several modules, while structures defined by Almeida‐Neto *et al*. (2008) as ‘beta‐diversity’ and ‘exclusive subsets’ have as many modules as plant nodes. Felix *et al*. (2022) have, however, shown that modularity and nestedness can coexist when compound topologies structure the network. The main point is that, in many cases, including this work, the observed level of nestedness (regardless of the direction) and modularity can be interpreted as two ways of quantifying the same type of topological structure. In other words, in our case, modularity can induce anti‐nestedness.

Because we found a consistent pattern in plant–AM fungi association networks, it is then natural to ask how anti‐nestedness and modularity emerge and what the ecological implications are. We cannot resolve this question with the data at hand. But we can offer some hypotheses, based on the strong foundation of a statistically robust structure, that allow us to propose future directions of research. The first observation is that anti‐nested networks can be dynamically stable (Staniczenko *et al*., 2013), meaning there could be an eco‐evolutionary advantage in the emergence of this structural property in the network. To test this hypothesis, perturbation experiments should be conducted in a set of plant–AM fungi assemblages along a gradient of anti‐nestedness or, conversely, nestedness. Results, as the ones presented here, practically show how those gradients can first be detected in natural communities, which could then be used to conduct manipulative experiments. Related to that, and in terms of mechanisms of origin, one hypothesis is that anti‐nestedness in plant–AM fungi association networks could emerge from the balance between mutualism and competition in interaction networks (Husband *et al*., 2002) or the balance in the mutualism–parasitism continuum. For example, one more specific hypothesis is that competition between both plant and AMF species may shift communities from generalist to specialist associations (Ricciardi *et al*., 2010) so as to generate the niche partitioning required to stabilise coexistence. That is a very specific process; however, that will require dedicated experiments and also metrics (e.g. the specialisation metrics proposed by Blüthgen *et al*., 2006). Also, more broadly, the processes at play must be more complex than just niche partitioning because the multiple scales and levels of aggregation at which we consistently observed anti‐nestedness and modularity obviously imply a number of factors at play. In glasshouse studies (Alguacil *et al*., 2011a; Dieng *et al*., 2015; Campos *et al*., 2018; Fernández *et al*., 2022) with good control of soil type, AMF species, host plants, moisture, and nutrient availability, specific AM fungi–plant combinations are likely to determine specialised modules between treatments in the experiments. In local field experiments, the AM fungi–plant associations occur in a more natural context under a range of specific, local biotic and abiotic conditions (Galván *et al*., 2009; Öpik *et al*., 2009; Tchabi *et al*., 2009; Wilde *et al*., 2009; Wang *et al*., 2011, 2024; Li *et al*., 2014; Grilli *et al*., 2015; Varela‐Cervero *et al*., 2015; Moora *et al*., 2016; Higo *et al*., 2020; Lara‐Pérez *et al*., 2020; Djotan *et al*., 2023; Garrido *et al*., 2023). Our hypothesis, which needs future testing, is that in those cases the high modularity in plant–AM fungi association networks arises from a response to environmental heterogeneity and, possibly, also from dispersal limitations (Paz *et al*., 2021; Davison *et al*., 2015). Finally, in the case of continental studies (Baar *et al*., 2011; Davison *et al*., 2011; Moora *et al*., 2011; Polme *et al*., 2016) that encompass diverse climates, soil types, and geographic features, high modularity simply results from large‐scale biogeographical and climatic differences in biota, which group by regions. Environmental gradients over large distances are expected to select plant and AM fungi associations, leading to modular structures and, indeed, anti‐nestedness because communities with fewer species are not a subset of communities with more species. Over large distances, limited dispersal and local adaptation of plants to native AM fungi can also strengthen modularity simply because similarity in the associations within a region will be higher than between regions. Our study also includes global‐scale studies (Davison *et al*., 2015, 2018; Zhao *et al*., 2021), which can be explained along the same lines. Overall, the conclusion we can make is that the consistency of our network patterns across a range of scales implies that multiple factors and processes contribute to nestedness/anti‐nestedness and modularity at multiple scales and levels of biological organisation, and only dedicated manipulative experiments at local scales or well‐designed surveys coupled with modelling can tease apart the factors causing nestedness/anti‐nestedness and modularity in the associations. Such approaches would also enable assessment of the effect of network structure on features such as the stability of the associations, for example, in response to perturbations.

Our null model results provide some direction for future investigations: In almost all cases, the model indicated that the observed level of anti‐nestedness and modularity highly deviated from the null distribution, meaning that the degree sequence *per se* is not a predictor of the two high‐level network features we investigated. In other words, aspects of node identity other than the degree must play a significant role in shaping the network or at least the two features we examined in this work (Caruso *et al*., 2022). While that result cannot definitively identify the causes that make plant–AM fungi associations anti‐nested and modular, it does offer compelling evidence that the key factors are related to the identity of the nodes in the network rather than the ability of a node to associate with a high or low number of other nodes. Therefore, the processes that structure plant–AM fugni associations must be researched within the eco‐evolutionary dynamics that generate diversity between nodes (e.g. plant species or populations and their symbionts). These will most likely include interactions such as competition within the root, environmental heterogeneity that may allow plant coexistence at broadscales, and selectivity in plant–AM fungi associations that can modulate that coexistence, as well as plant response to environmental gradients. In other words, the biological and ecological identities of the nodes matter both for plants and AM fungi and, more specifically, it determines anti‐nestedness and high modularity in the investigated networks, given the null model results.

Future research will have to identify what specific aspects of node identity matter. One promising avenue for that is community phylogenetics (Webb *et al*., 2002; Cadotte & Davies, 2016): Past studies, including some of ours, have already shown that AMF community display non‐random phylogenetic patterns (Horn *et al*., 2014, 2017), which have in some cases been linked to phylogenetically conserved traits (Powell *et al*., 2009). The same applies to plants, and there are examples of bipartite network analysis linking plants and their fungal symbionts in the context of the phylogenetic correlation between the two groups (see Jacquemyn *et al*., 2011, especially their Fig. 2). In the context of maximum entropy networks, the way to introduce phylogeny into the network model is the formulation of the node‐level constraints accounting for the phylogenetic correlation between nodes, which would create blocks of nodes in a layer that tend to be more connected to certain blocks of nodes in the other layer. In other words, our *p*_ij_, or probability of connection between node *i* and *j*, would be recalculated to account for phylogenetic correlations between *i* and *j*, which would imply updating association structures in the null model random matrices and, therefore, an updated null model distribution and, finally, potentially different outcomes. The specific analytic formulation of this type of maximum entropy network is a future avenue of theoretical research (Caruso *et al*., 2022).

In conclusion, we are confident that our description of network patterns is particularly robust given our modelling premises and the nature of the data at hand. We propose that the next generation of network models applied to any plant–microbial interaction should thus embrace soft constraints and their fluctuations in network models. Here, however, we only focused on the degree sequence and a binary configuration model, as that was the main focus of past studies, and quantitative information on the links is scarce or debated. Apart from phylogenetic correlations, our modelling framework is straightforward to extend to quantitative networks with weighted links (Squartini & Garlaschelli, 2017), something that traditional rewiring models are struggling with (Caruso *et al*., 2022). In that case, the first property to consider in the future is the strength sequence, which is the sum of the weights of all links to a node. Weighted links quantify the strength of associations between two nodes or can also describe fluxes between nodes if the network links are directed rather than undirected. Yet, the quest to quantitatively characterize the strength of association between AM fungi and plants remains a challenge, with the exception of highly mechanistic studies investigating fluxes of matter between plants and fungi (Lekberg *et al*., 2024). The network models to analyse weighted links already exist, and future research will have to identify robust methods to quantify the strength of association between plant and fungal network nodes, which will represent a significant step forward in our understanding of these associations.

## Competing interests

None declared.

## Author contributions

SA and TC conceptualized and designed the manuscript. SA gathered the literature., SA and NA ran the network analysis. SA, AL‐G, HB, MP‐M and TC designed and finalized the figures. AL‐G, JLG, JMA and MP‐M performed the aggregation of Garrido *et al*. (2023) dataset. SA organized and structured the information, ran the analyses, and wrote the manuscript with TC. All authors (SA, NA, AL‐G, HB, MP‐M, LL, JL‐G, JM‐A, MC‐R, DJ and TC) reviewed and contributed to the text of the manuscript.

## Disclaimer

The New Phytologist Foundation remains neutral with regard to jurisdictional claims in maps and in any institutional affiliations.

## Supporting information


**Dataset S1** Exemplar dataset used to fit the maximum entropy model.

**Fig. S1** Prisma Flow Diagram, a detailed workflow for literature review and selection of studies for network analysis.

**Notes S1** Annotated R code used to fit the maximum entropy model.

**Table S1** Detailed summary of selected studies, metadata associated with these studies, detailed results of network parameters calculated, information on plant nodes used to construct network models and the results of network parameters acquired via aggregation of a detailed study that is Garrido *et al*. (2023).
Please note: Wiley is not responsible for the content or functionality of any Supporting Information supplied by the authors. Any queries (other than missing material) should be directed to the *New Phytologist* Central Office.

## Data Availability

The data presented in this study are already publicly available as per the references given in Table S1 (S4).
