# Supplementary figures and images for: Maximum entropy networks show that plant–arbuscular mycorrhizal fungi associations are anti‐nested and modular
Source: New Phytol. 2025 Nov 9;249(1):460–75. doi: 10.1111/nph.70694 (PMC12676088; doi:10.1111/nph.70694)

## Identification of Plant AMF association studies via databases and Web Resources

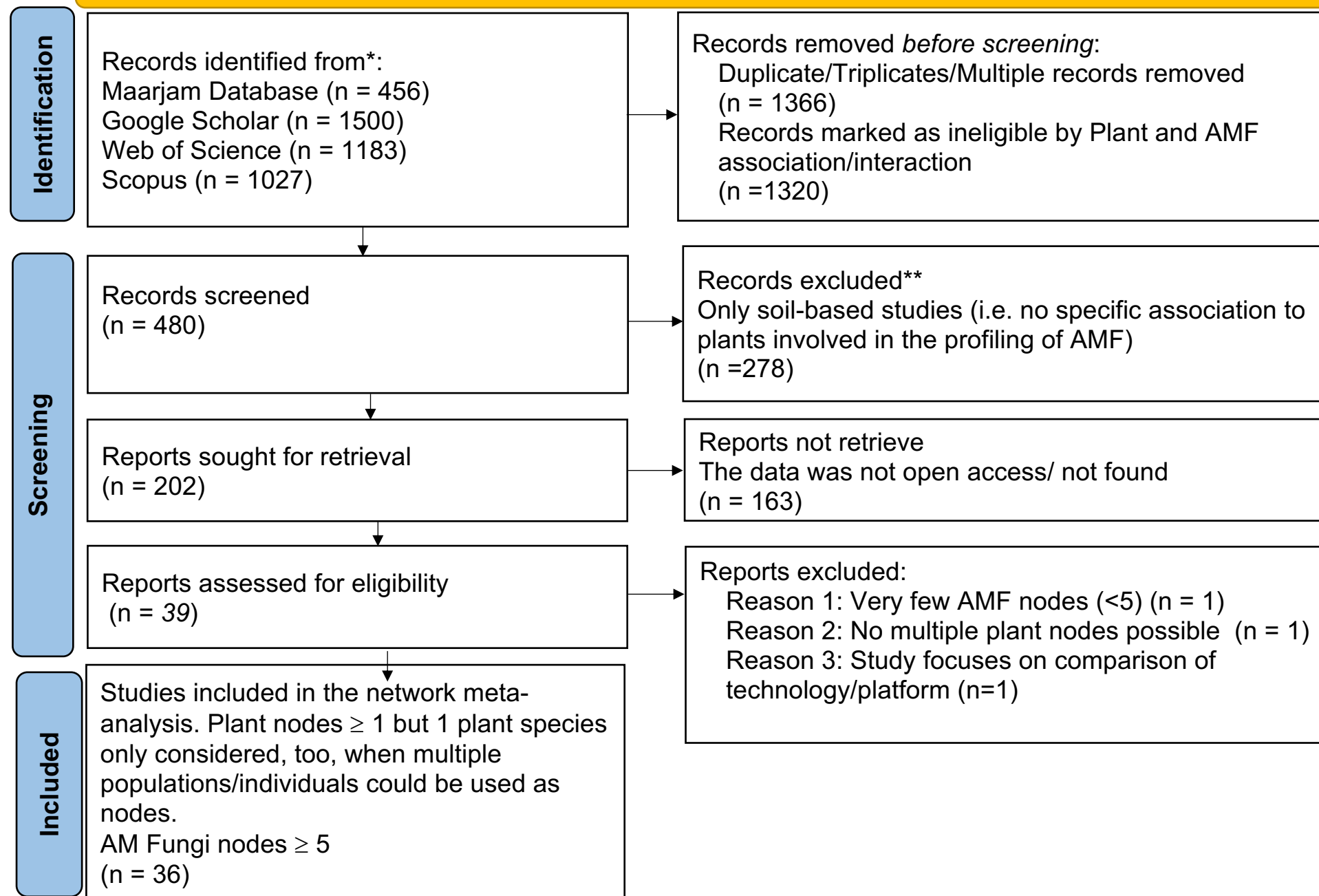

Supplement: Supplementary file 2 — Fig. S1 Prisma Flow Diagram, a detailed workflow for literature review and selection of studies for network analysis. [file NPH-249-460-s003.pdf]
